# A Facile and Efficient Bromination of Multi-Walled Carbon Nanotubes

**DOI:** 10.3390/ma14123161

**Published:** 2021-06-08

**Authors:** Sandra Zarska, Damian Kulawik, Volodymyr Pavlyuk, Piotr Tomasik, Alicja Bachmatiuk, Rafał Szukiewicz, Wojciech Ciesielski

**Affiliations:** 1 Faculty of Science and Technology, Institute of Chemistry, Jan Dlugosz University in Czestochowa, 42-200 Czestochowa, Poland; sandra.zarska@doktorant.ujd.edu.pl (S.Z.); d.kulawik@ujd.edu.pl (D.K.); v.pavlyuk@ujd.edu.pl (V.P.); 2 Nantes Nanotechnological Systems, 59-700 Boleslawiec, Poland; rrtomasi@cyf-kr.edu.pl; 3 Polish Center for Technology Development, Laboratory of Electron Microscopy and Material and Geological Analysis, Lukasiewicz Research Network, 54-066 Wroclaw, Poland; alicja.bachmatiuk@port.lukasiewicz.gov.pl; 4 Faculty of Physics and Astronomy, Institute of Experimental Physics, University of Wroclaw, 50-204 Wroclaw, Poland; rafal.szukiewicz@uwr.edu.pl

**Keywords:** MWCNT, bromination, functionalization, X-Ray photoelectron spectroscopy

## Abstract

The bromination of multi-walled carbon nanotubes (MWCNT) was performed with vapor bromine in a closed vessel, and they were subjected to intensive stirring with a magnetic stirrer for up to 14 days. The efficiency of bromination was compared depending upon duration. The structure and surface of the crude and purified products were characterized by detailed physicochemical analyses, such as SEM/EDS, TEM, XRD, TGA, Raman, and XPS spectroscopies. The studies confirmed the presence of bromine covalently bound with nanotubes as well as the formation of inclusion MWCNT–Br_2_ complexes. It was confirmed that Br_2_ molecules are absorbed on the surface of nanotubes (forming the CNT-Br_2_ complex), while they can dissociate close to dangling bonds at CNT defect sites with the formation of covalent C−Br bonds. Thus, any covalent attachment of bromine to the graphitic surface achieved around room temperature is likely related to the defects in the MWCNTs. The best results, i.e., the highest amount of attached Br_2_, were obtained for brominated nanotubes brominated for 10 days, with the content of covalently bound bromine being 0.68 at% (by XPS).

## 1. Introduction

Carbon nanotubes (CNTs), which were discovered in 1991 by Iijima [1], evoke considerable interest, with numerous fundamental studies and practical applications. For their morphology, CNTs are classified into SWCNT (single-walled), DWCNT (double-walled) and MWCNT (multi-walled) nanotubes. Structurally, MWCNT have multiple rolled up graphite layers which exhibit different interactions. Furthermore, MWCNT have a lot more defects (structural, interstitial sites, gaps) than SWCNT, which has a significant impact on their physicochemical properties [2]. Their dispersal in organic solvents and water is difficult. Native CNTs are chemically inactive and the presence of carbon atoms of sp^2^ hybridization in hexagonal networks prevents the formation of chemical bonds with surrounding molecules [3]. The solution to this problem is to modify the surface properties of carbon nanotubes. It is possible in two ways: (i) covalent attachment of chemical groups to tube ends or sidewalls, particularly in the sites of structural defects [4], and (ii) non-covalent interactions with functional molecules leading to the formation of supramolecular complexes [5,6,7,8,9,10,11,12].

The intrinsic mechanical and transport properties of CNT, which result from their structure, make them the ultimate carbon fibers, combining stiffness, strength and tenacity. When admixed to several materials, e.g., polymers (polystyrene, epoxy resin, polyvinyl alcohol, poly(methyl methacrylate), they considerably reinforce their mechanical properties [13,14]. Additionally, the thermal and electrical conductivity of CNT are not comparable to other conductive materials, such as graphite, diamond, steel or cooper [15].

The functionalization of CNTs by controlled structural changes is crucial for its applicative significance. Attaching functional groups or introducting heteroatoms to the CNT structure can increase dispersion and processing capabilities [16,17,18] and tailored physical and chemical properties [19,20] to improve the mechanical, thermal and optical properties of the CNTs [21] in a wide range of applications in material [22] and biomedical sciences [20,23,24]. Moreover, CNTs are frequently doped with heteroatoms, such as N, B, P, S, Si and Se to modify the chemical properties of the carbon material. The introduction of these atoms increases or decreases reactivity, which results from the excess or deficiency of electrons in the structure, and changes conductivity as well [25,26,27,28,29].

It is necessary to use special halogenation methods due to the significant chemical passivity of the CNT surface, especially for bromination because of low-reactive bromine atoms. On the other hand, bromine derivatives of CNTs, in comparison to fluoro or chloro derivatives, are more reactive compounds, and therefore more desirable in carrying out further functionalization [30]. The bromine atom in the C–Br bonds in CNTs readily undergoes nucleophilic substitutions. Hence, the functionalization of CNT leading to the production of their brominated derivatives opens a path for introducing numerous functional groups to CNTs.

So far, known methods of bromination of MWCNTs require rather demanding (conducted mostly at high temperatures) conditions for the reaction, and are described in detail in our review [31]. The bromination was performed by electrochemical [32] and microwave methods [33] under UV light irradiation [34], or with a set of Lewis acids [35] with bromine in the liquid [36], gas [30,37] and plasma [38] phases. Bromination can be useful in the purification of commercially available CNT, which usually contains the catalyst used for their growth with amorphous carbon. Among the various solutions [2], CNTs can be successfully purified with a bromination method that involves either one-step or multi-step methods [39,40,41,42]. As reported, the multi-step purification process of MWCNTs consisted of ultrasound, heat treatment in hot water, bromination, oxidation and acid treatment. It was shown that bromination played a key role in purifying CNTs and that the purity of material after halogenation was above 94% with an efficiency of about 50%, while the content of iron particles approached 2 wt% [43]. The strong catalytic influence of Br-containing CNTs were studied and the possible use of MWCNT-Br as active additives in the oxidation of alkylaromatic hydrocarbons was recommend by Zeynalov et. al. [44,45,46]. By using the bromination reaction, further functionalization of the material surface is possible, e.g., surface-grafted bromine can be replaced on the diethylamine groups [37]. It is known that the metallic nanotubes are more reactive to Br_2_, while the semiconducting nanotubes also readily interact with bromine [47].

In this paper, the chemical modification of MWCNT surfaces with Br_2_ vapor using magnetic stirring at 30 °C for different time periods has been presented. Our method was simple, because it did not require the use of complicated, expensive devices (MW, plasma), only basic laboratory equipment. In addition, it was carried out at a temperature slightly above room temperature, in mild conditions, which allowed for low energy consumption and made the process more environmentally friendly. The applied method confirmed that the covalently linked bromine with multi-walled carbon nanotubes was obtained. The developed method of bromination as a simple way of increasing the reactivity of native MWCNTs allows for their further modification and a significant improvement in their application properties.

## 2. Materials and Methods

### 2.1. Materials

All reagents were of analytical grade. They were purchased from Chempur/Sigma-Aldrich (Piekary Slaskie, Poland). MWCNTs NC7000 were manufactured by NANOCYLTM (Sambreville, Belgium), which were produced through the catalytic chemical vapor deposition (CCVD). Their average diameter and length were 9.5 nm and 1.5 µm, respectively. They consisted of 90% carbon and <1% metal oxide and had a surface area of 250–300 m^2^ g^−1^ [48].

### 2.2. Bromination of MWCNTs

**Figure 1:**
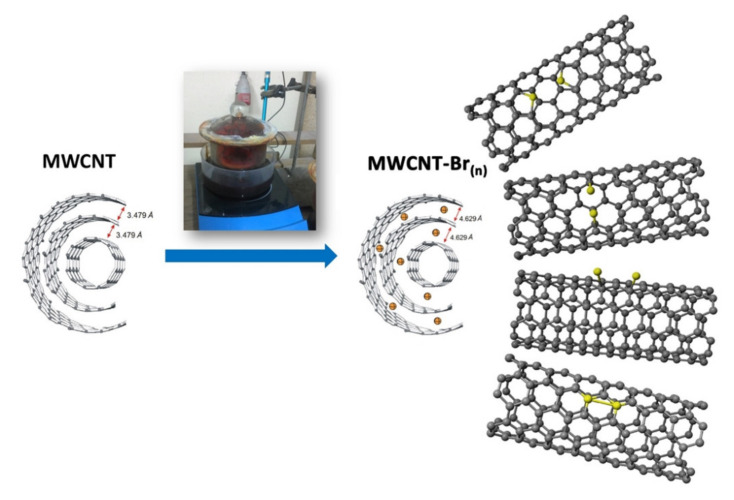
A schematic representation of the experimental setup.

Native MWCNTs (~20 mg) were placed on a glass Petri dish and closed in a vessel with liquid bromine (~50 mL). The intense magnetic stirring lasted 7, 10 and 14 days, to increase the vapor pressure of bromine in the vessel. The vessel was placed in an oil bath and heated at 30 °C (Figure 1). After that period, the crude product was purified in accordance with the method of Drabowicz, Ciesielski and Kulawik [49]. The bromine vapors were blown from the vessel into a sealed container containing sodium thiosulfate for quick and safe neutralization. Then, they were washed with distilled water and centrifuged at 14000 rpm until of neutral pH. Subsequently, methanol was added to the residue and centrifuged. The residue was placed in the oven for at least 12 h at 65 °C. Finally, benzene (10 mL) was added to the sample to bind unreacted bromine and it was centrifuged again. Then, the benzene was decanted, and the residue was maintained for at least 12 h in a drying oven at 80 °C. 

### 2.3. Characterization Methods

The surface morphology of the sensing area of native and brominated MWCNT was examined using a Nova Nano SEM 200 microscope of up to 2 nm resolution and 70–500,000× magnification equipped with a field FEG Schotky emitter (FEI Europe Company, Eindhoven, Netherlands). Samples were used in the powder forms and were subjected to a beam energy of 18 kV.

For chemical analysis in microareas by energy dispersive X-ray spectroscopy (EDX), a Tescan Vega 3 SBU scanning electron microscope (SEM) (Tescan, Brno, Czech Republic) with an WDS/EDS analyzer was used.

The samples were characterized using transmission electron microscopy (TEM) on a Titan^3^ G2 60-300 microscope (FEI, Hillsboro, United States) with an accelerating voltage of 80 kV.

X-ray photoelectron spectroscopy (XPS) was used to determine the surface composition of analyzed samples. The PREVAC 426 system configuration, equipped with a SCIENTA R3000 (Scienta Omicron, Uppsala, Sweden) hemispherical photoelectron spectrometer and monochromatic Al Kα source operating at 450 W was used. The overall resolution of the spectrometer during measurements was 0.7 eV as a full width of half of the maximum (FWHM) of the Ag3d_5/2_ line. The base pressure in the analysis chamber was greater than 3 × 10^−10^ mbar. D-Auger parameters for all samples were determined and all acquired spectra were calibrated to sp^2^ bond at 284 eV [50]. After the subtraction of the Shirley-type background, the core-level spectra were decomposed into main components with mixed Gaussian–Lorentzian lines (70% G + 30% L for majority of photo-peaks) by a non-linear least squares curve-fitting procedure, using CasaXPS software.

The shredded sample was stuck to the glass fiber using glue. The measurement was done by X-ray diffraction (XRD) of single carbon fibers. The data on carbon fibers were collected at room temperature using a four-circle diffractometer (Xcalibur Oxford Diffraction, Abingdon, UK) with a CCD detector (graphite monochromatized Mo-Kα radiation), and the ω-mode scans were performed. The analytical absorption corrections were made by CrysalisRed [51]. The structures were solved by direct methods and refined by full-matrix least-squares procedures using the SHELX-97 software package [52]. An initial parameter set was obtained from the automatic interpretations of direct methods using SHELXS-97, and this structure model was further refined until convergence was reached using SHELXL-97. X-ray diffraction on powderized samples was performed by means of a diffractometer URD-6 with a linear PSD detector for Cu-Kα radiation. Additionally, the structure the model received from single crystal methods was also refined from powder diffraction data with the program FullProf from the WinPLOTR software using a pseudo-Voigt profile function [53,54].

The Raman spectra were recorded in a backscattering configuration on a Renishaw InVia spectrometer (Renishaw plc, Wotton-under-Edge, UK) using a 532 nm diode laser (diode-pumped, frequency-doubled Nd:YAG). The spectra were gathered with 10% of laser power (2.9 mW).

The weight loss steps (thermogravimetry, TG) and thermal effects (differential scanning calorimetry, DSC) were measured using a DSC–TG–DTG NETZSCH STA 409C apparatus (Netzsch, Selb, Germany). The thermal decomposition was studied over a temperature range of 20 °C to 900 °C at a heating rate of 5 °C min^−1^ under static air conditions. Additionally, all the samples (about 20 mg) were heated in corundum crucibles with non-hermetic lids. Furthermore, the recorded thermograms were analyzed with the NETZSCH–TA–ANALYSIS.

## 3. Results and Discussion

**Figure 2:**
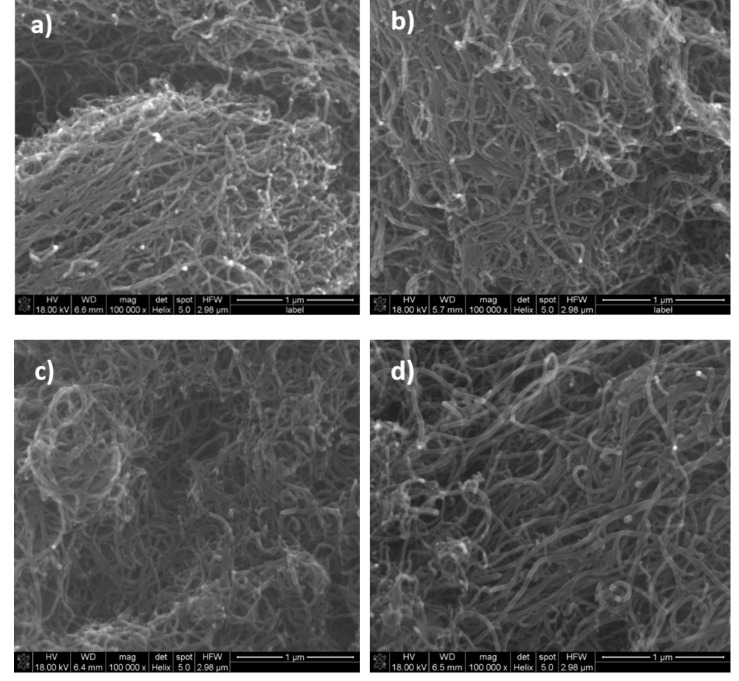
The SEM images of MWCNTs: native (**a**) and brominated for 7 (**b**), 10 (**c**) and 14 days (**d**).

The SEM images of native and brominated MWCNT samples are shown in Figure 2. One can see that the bromination influenced the nanotube structure. The bromination resulted in a slight increase in the nanotube diameters (compare, for instance, Figure 2a,d). The comparison of the images indicates that after bromination, the average diameter of MWCNT ropes is slightly increased and more separated nanotubes become visible. The SEM images (Figure 2b–d) indicate changes in the morphology of MWCNTs with bromination and, notably, a detectable increase in the interlayer separation, which would be expected if Br_2_ molecules intercalated between the inner and outer tubes.

**Figure 3:**
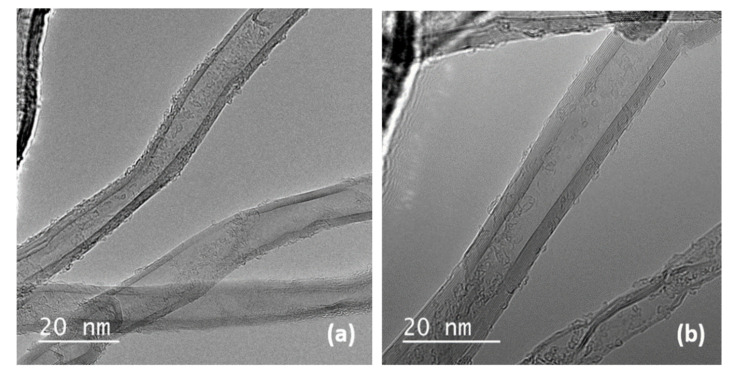

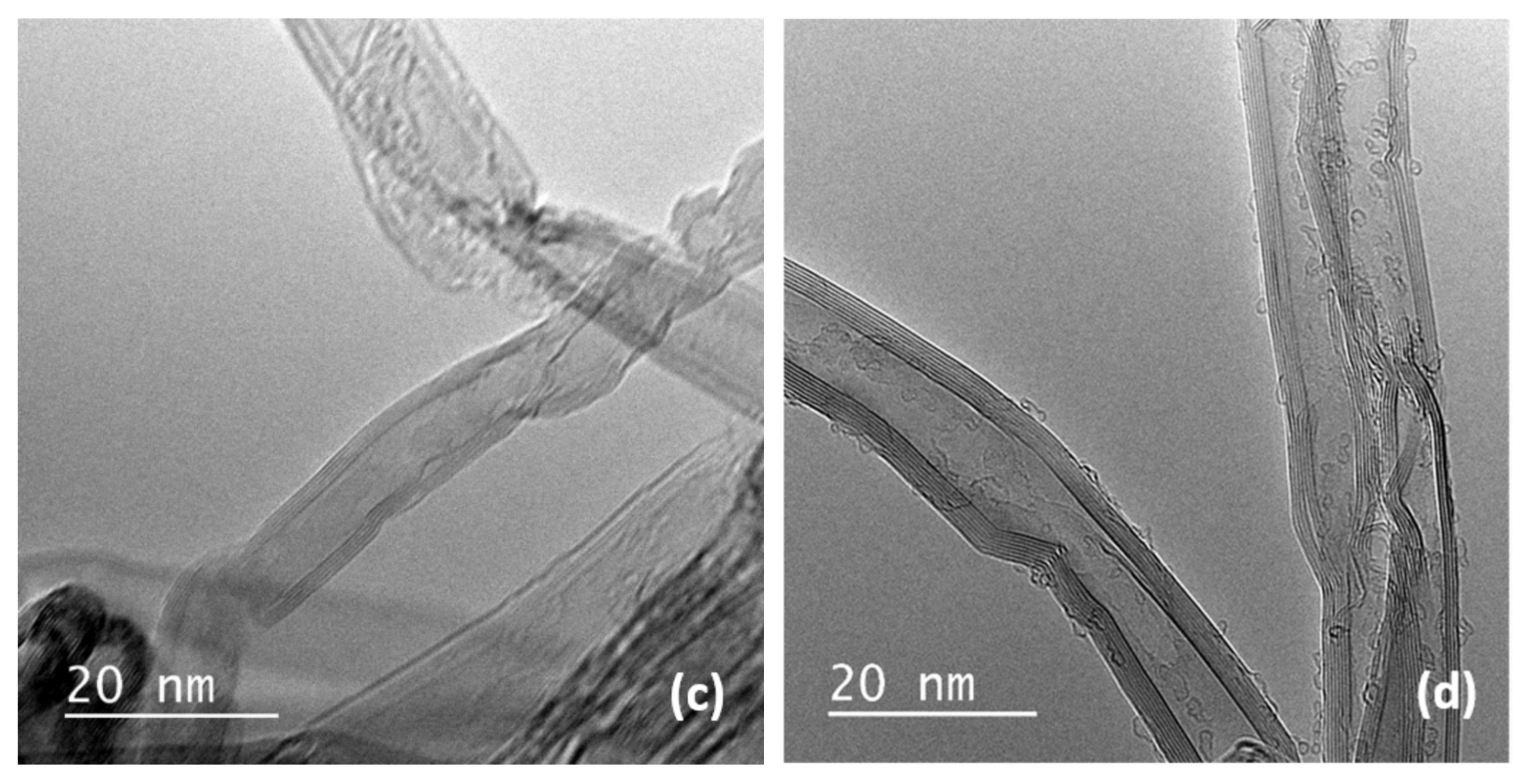
TEM images of MWCNTs: native (**a**) and brominated for 7 (**b**), 10 (**c**) and 14 days (**d**).

The structure and morphology of initial and brominated MWCNTs were also investigated using transmission electron microscopy (Figure 3). No significant changes were observed for the brominated sample, thus indicating that bromine treatment at moderate temperatures inflicts little damage on the nanotube structure after bromination for 7 (Figure 3b), 10 (Figure 3c) and 14 days (Figure 3d). Analysis was performed for agglomerates of nanotubes revealing 0.8–2.4 wt% of bromine.

The chemical analysis in microareas of completely purified crude product carried out with energy dispersive X-ray spectroscopy showed that the product after 10 days bromination contained 2.4 wt% chemically bound bromine (Table 1). It was the highest content of bromine found in the prepared bromination products. This result will be analyzed later.

The surface composition of the selected samples was investigated with X-ray photoelectron spectroscopy. The total concentrations of atoms in the samples were determined on the basis of survey spectra, taking into account the presence of elements C, O, Br.

Data for all samples are presented in Table 2. For all samples, D-Auger parameters have been determined from differentiated C KLL Auger peaks, which indicate the presence of sp^2^ bonds. Obtained values are in good agreement with literature data, e.g., for graphite (21 eV) and for diamond (13 eV) [50]. In Table 1, the data from EDX measurements are related to the sample volume data, while the data in Table 2 are XPS data connected to surface measurements up to a few nanometers from the surface. This means that the depth of the medium’s (electrons, photons) penetration is different, and thus the obtained values of the samples’ elemental composition may vary.

**Figure 4:**
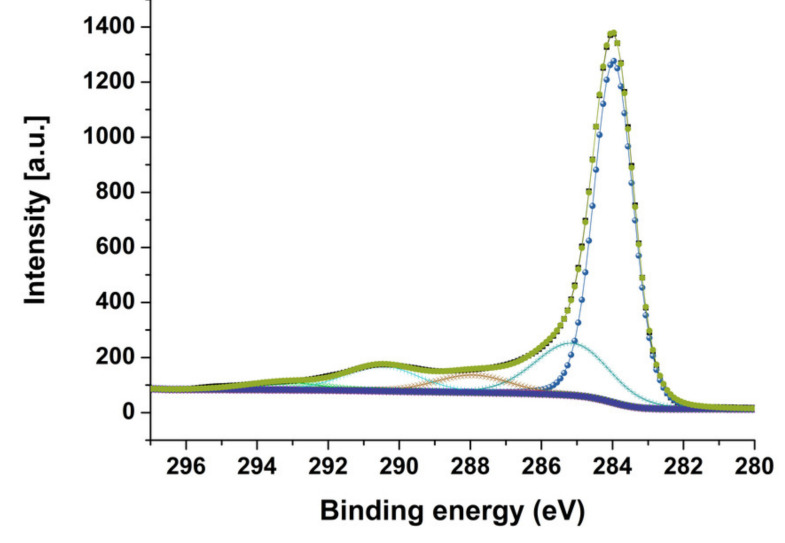
High resolution C1s regions of MWCNT–Br 10 sample.

High resolution C1s regions from sample MWCNT–Br10 shown on Figure 4 consist of the following components: sp^2^ (284.0 eV), sp^3^ (285.0 eV), C–O/C–Br (286.4 eV), C=O (288 eV), O–C=O (289–290 eV), and shake-up satellites at around 292 eV. C1s regions from other samples look similar, which is why percentage carbon species concentration are presented for comparison in Table 3.

**Figure 5:**
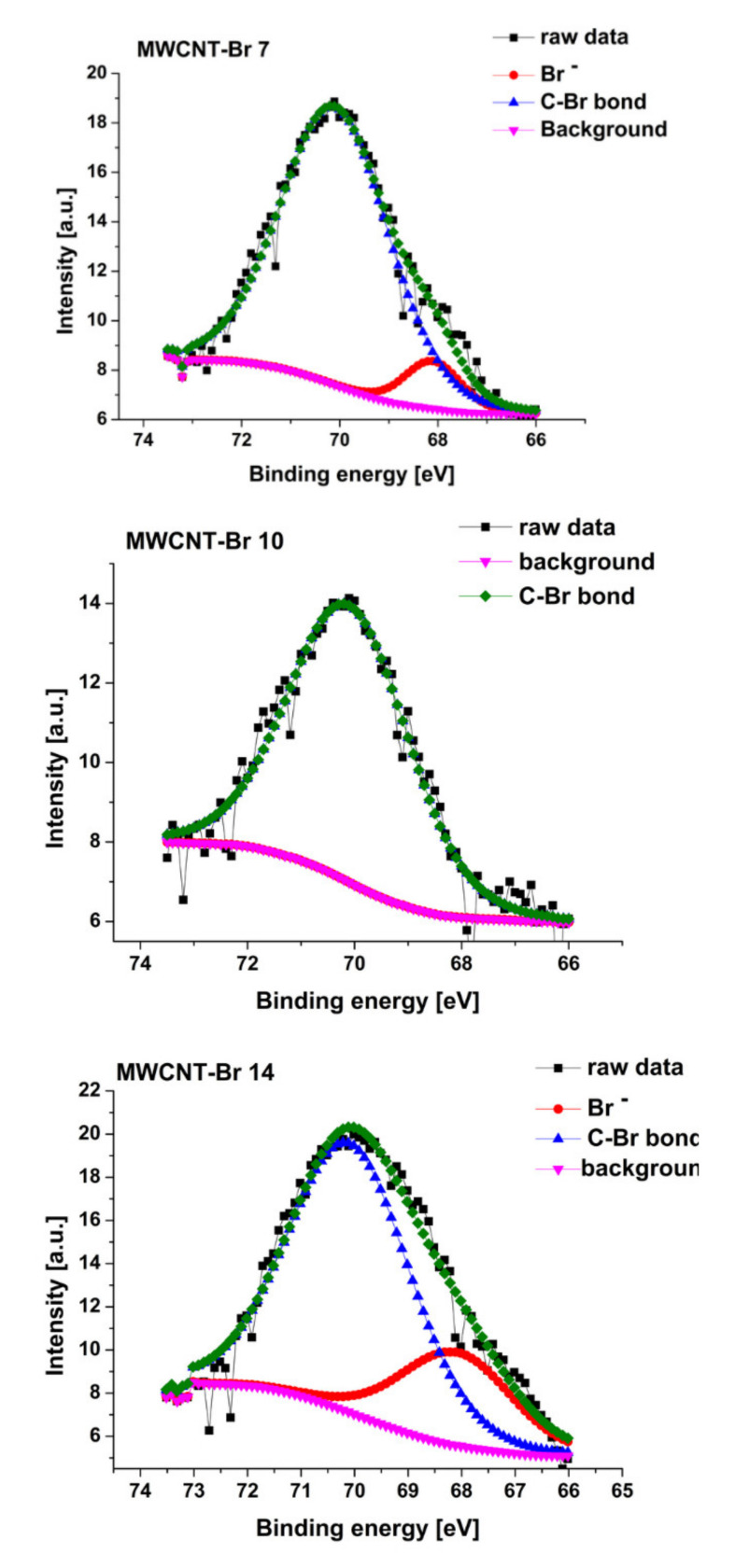
Br3d region for samples after the bromination process.

The process of bromination of multi-walled carbon nanotubes leads to the formation of C-Br bonds. The Br3d region in brominated samples was decomposed into two components with energies (70 eV) for covalently bonded Br atoms C-Br and negatively charged Br species (67.9 eV) (Figure 5) [30,55]. One should note that the bromination process of multi-walled carbon nanotubes leads not only to the formation of C-Br bonds but also of other species such as C=O and O–C=O. The bromination process of multi-walled carbon nanotubes leads not only to the formation of C-Br bonds but also of other species such as C=O and O–C=O. It should be noted that in the case of total atomic concentration (Table 2) for all samples, the MWCNT-Br 10 sample is different. In Table 2 there is a clearly observed increase in oxygen concentration. Such a situation also confirms the EDX data (Table 1) of a strong increase in Br content in a sample volume and a decrease for the MWCNT-Br 14 sample. Obtained data from C1s regions (Table 3) confirms that in the case of the MWCNT-Br 10 sample there is a decrease in C-O/C-Br bonds instead of an increase in other C=O and O–C=O species. Such a situation is not observed for the other samples. This situation should be connected with the bromination process.

**Figure 6:**
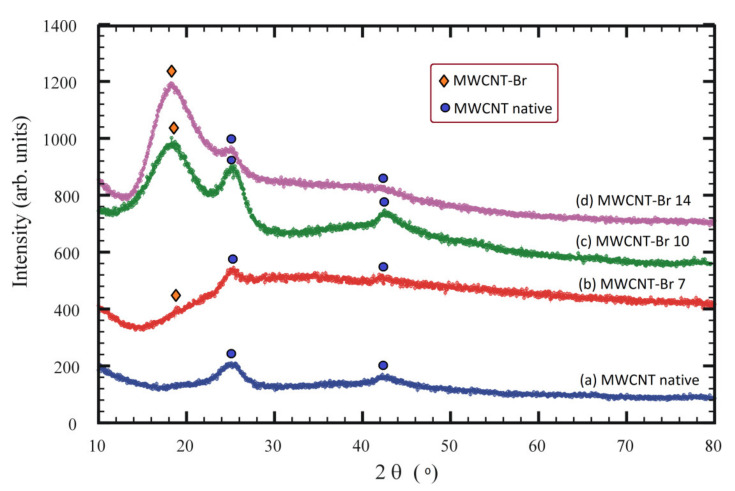
XRD patterns of native (**a**) and brominated MWCNT (**b**–**d**). The positions of diffraction peaks are marked as blue circles for native and orange rhombuses for brominated MWCNT, respectively.

The diffraction pattern (Figure 6a) is typical for native MWCNT, which is characterized by peaks at 25° and 42°. The diffraction patterns (Figure 6b–d) show that brominated MWCNT samples are a mixture of native MWCNT and MWCNT-Br. For MWCNT-Br there is a characteristic peak at 18°. Therefore, increasing the bromination time for the samples b-d increases the intensity of this peak at 18°, which indicates an increase in the amount of bromination in this phase. The interlayer distance in native MWNCT estimated from the X-ray data (Figure 6a) was 3.479 Å. The shift of the diffraction peaks toward the lower 2θ angles for the bromine modified phase (MWCNT-Br) indicates an increase in the distance between the layers.

**Figure 7:**
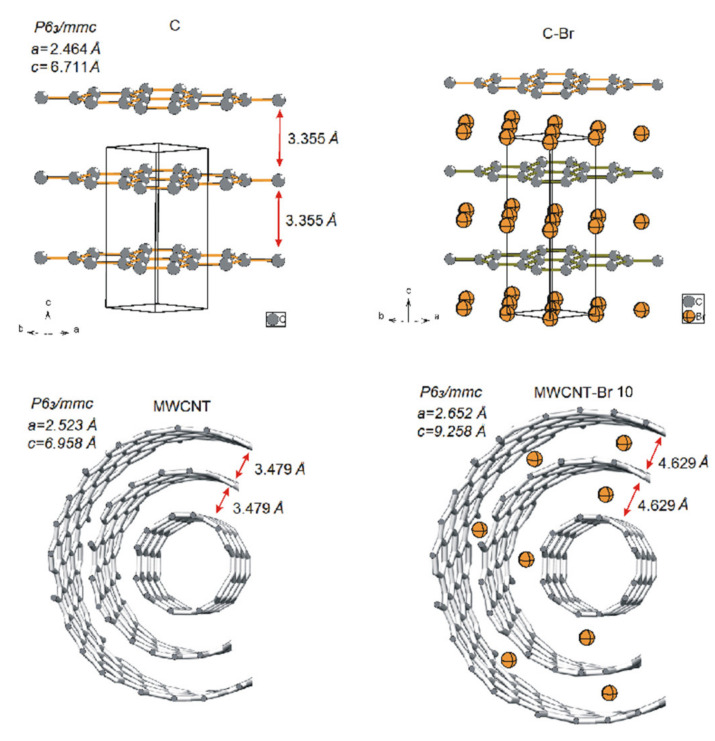
Model structure of native and 10-day-brominated MWCNT.

The X-ray diffraction pattern of the brominated nanotubes for 7, 10 and 14 days, respectively, showed that the distances between these layers rose to 4.310, 4.629 and 4.710 Å, respectively (Figure 7, Table 4). Furthermore, the increase in these interlayer distances, which is caused by the interlayer inclusions of bromine atoms and consequently, the shift in reflections, clearly points to the possibility of the modification MWCNTs in that manner. One might see that with prolonged bromination time, MWCNT swells as a result of an expansion in the C–C bonds and interlayer distances, with the space group remaining unchanged. Additionally, that expansion could explain the highest yield of bromination being found on the 10th day of the process and its declined yield on the 14th day. Due to an increase in the a-parameter (unit cell dimensions), the molecular π-orbitals of particular tubes diffused, overlapping the orbital with the molecular orbitals of the bromine molecules in the transition complex, which led to the covalently bound bromine atoms becoming weaker. Additionally, the expanded interlayer distances contributed to the weaker bonding of the bromine molecules in the transition state. Both factors removed the bromine molecules from the crude product upon its purification by extended washing.

**Figure 8:**
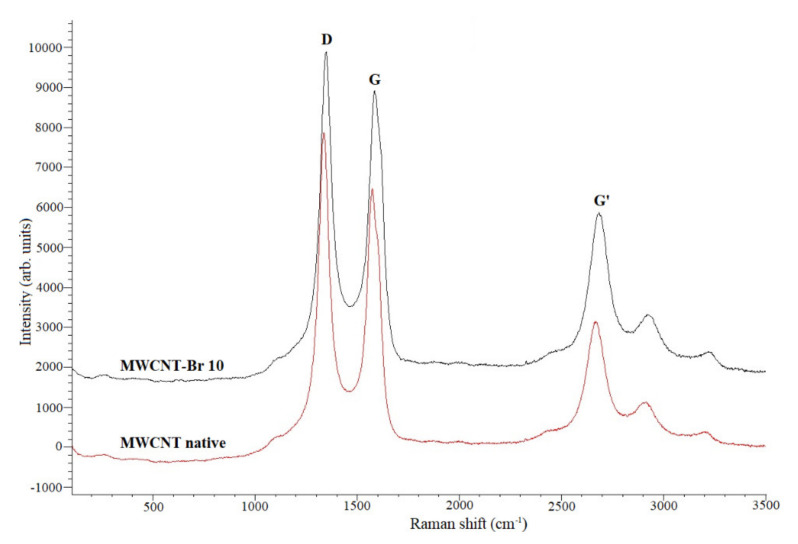
Raman spectra for native MWCNT and MWCNT–Br 10.

In the Raman spectra (Figure 8) of MWCNTs, the three bands D, G and G’ could be distinguished. The two first bands are particularly important for the evaluation of the consequences of the modifications of MWCNTs. They reflect the vibrations of carbon atoms along the axis of MWCNTs. Additionally, the position of the band D is associated with the arrangement of the structure and thus with the possible defects in the carbon network, while the G band is characterized by the structure of graphite. The G’ band informs the disorder symmetry and thus the sp^2^ hybridization. Band D’, which is derived from the vibration perpendicular to the walls of the nanotubes, is not always observed because it overlaps with the G band. The Raman shifts for MWCNT prior to and after synthesis bromination (Table 5) allow for the formulation of the following conclusion. The observed shifts of the Raman signals of the D and G bands prove the rearrangement and change in the type of hybridization, and thus indicate the formation of covalent bonds.

**Figure 9:**
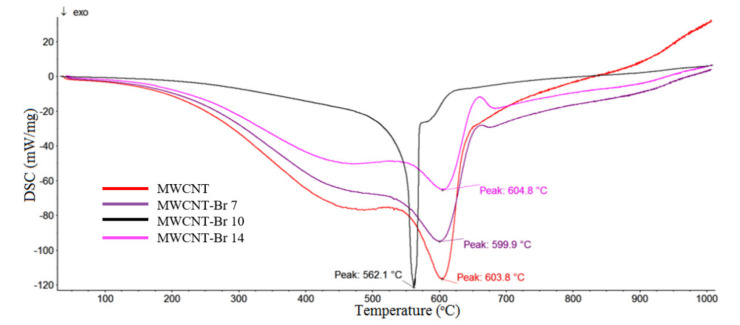
DSC curves for native and brominated MWCNTs.

**Figure 10:**
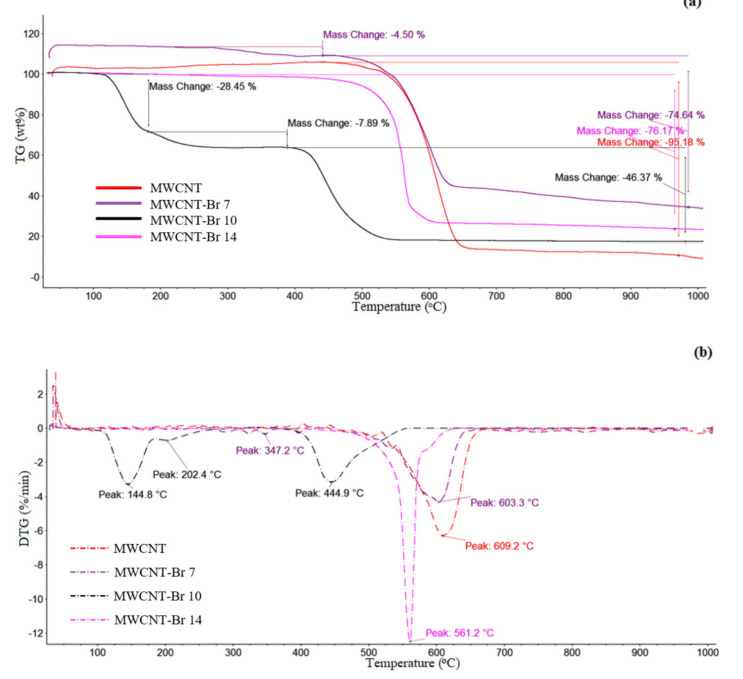
(**a**) Thermogravimetric (TG) and (**b**) differential TG (DTG) curves for native and brominated MWCNTs. The insert shows the thermal behavior of the samples in the interval from 50 to 1000 °C.

In order to determine the thermal stability of MWCNT-Br and the amount of bromine bound to the nanotubes, the DSC, TG and DTG measurements were performed. DSC and TG analyses showed changes in the thermal stability of carbon nanotubes after their bromination depending on the time of bromination (Figure 9 and Figure 10). Observation of the DSC/TG curves indicated that the weight loss of native carbon nanotubes begins at about 450 °C. As temperature increased, unmodified carbon nanotubes lost mass continuously, with a maximum decomposition peak at 603.8 °C. Additionally, the thermal distribution of samples brominated for 7 and 14 days was similar to that of native nanotubes, with maximum decomposition peaks at 604.8 and 599.9 °C (Figure 9). The thermal decomposition of nanotubes brominated for 10 days was significantly different from the distribution of previously discussed materials. The maximum thermal effect was shifted toward lower temperatures, with a peak at 562.1 °C, which consisted of two thermal effects—wider, with a maximum at 562.1 °C, and narrower, with a maximum at 564.0 °C, which might indicate another way bromine binds to the nanotubes. Weight loss of the native MWCNT was observed between 450 and 675 °C, and was 95.18 wt%. The addition of bromine to the nanotubes caused their stabilization and increased their resistance to thermal decomposition. The loss of weight for MWCNT-Br varied between 76 to 83 wt%. For a sample brominated for 7 days, weight loss was first observed at 277 °C and was 4.5 wt%. The first peak of weight loss of the MWCNT brominated for 10 days was observed at approx. 145 °C, which corresponded to the oxidation of the nanotubes (about 28 wt%). The second peak of weight loss started at 202 °C and was about 8 wt%, which corresponded to the removal of Br_2_ molecules associated with nanotubes. The analysis of the SEM images indicates an increase in the space between individual nanotubes, which confirmed bromine intercalation within the nanotube structures. The wide peaks on the DSC curves suggest that bonds, formed at different locations on the MWCNT surface, can have different energies, and this explains the detachment of bromine over a wide temperature range (Figure 9). This corresponds to the detachment of the particles of bromine embedded in the space of nanotubes and bound covalently with nanotubes. Additionally, the results of the XPS analysis confirmed the existence of various types of bromine bonds with nanotubes.

## 4. Conclusions

The interaction of MWCNTs with saturated Br_2_ at medium temperature was studied using SEM/EDS, TEM, X-ray diffraction and TGA, Raman, and XPS spectroscopies. The XPS results confirmed the presence of covalently bonded Br atoms and negatively charged Br species after the bromination process. Additionally, the SEM analysis indicates that interaction with Br_2_ causes partial debundling of nanotubes, and this procedure could be used for MWCNT dispersion. As shown, MWCNTs can be successfully and relatively easy brominated in much milder bromination conditions which makes our method more environmentally friendly.

Furthermore, it is confirmed that Br_2_ particles are adsorbed on the surface of nanotubes (forming the CNT-Br_2_ complex), while they can dissociate close to dangling bond at CNTs defect sites with the formation of covalent C−Br bonds. Thus, any covalent attachment of bromine to the graphitic surface achieved around room temperature is likely to be related to the defects in the MWCNTs or to CNT graphitization. Moreover, covalently bonded bromine can provide the chemical activity needed for nanotubes to couple organic residues via substitution reactions. Furthermore, brominated nanotubes may be a potential candidate to replace commercially used brominated flame retardants, because they have some chemical similarities to them. Additionally, materials like MWCNT-Br have great potential in the field of electrochemical sensors and microelectronic devices.

## 5. Patents

The process of preparing the brominated multi-walled carbon nanotubes (MWCNT) containing bromine atoms and their purification method. Drabowicz, J.; Ciesielski, W.; Kulawik, D. Pat. EP3002253, 19 February 2015.

## Figures and Tables

**Table 1:** Chemical analysis in microareas by energy dispersive X-ray spectroscopy (EDX) (wt%).

Samples	C	Br
MWCNT–Br 7	98.2	1.8
MWCNT–Br 10	97.6	2.4
MWCNT–Br 14	99.2	0.8

**Table 2:** Atomic concentration (at%) and D-parameter (eV) from XPS survey spectra.

Samples	Br	O	C	D-Parameter
MWCNT native	0	1.32	98.58	21.8
MWCNT–Br 7	0.74	2.18	97.07	21.8
MWCNT–Br 10	0.68	3.95	95.36	20.0
MWCNT–Br 14	0.62	1.94	97.44	20.6

**Table 3:** Comparison of carbon species concentration data (at%) from C1s regions.

Carbon Species	MWCNT Native	MWCNT–Br 7	MWCNT–Br 10	MWCNT–Br 14
sp^2^ (284.0 eV)	64.2	62.6	60.2	63.8
sp^3^ (285.0 eV)	15.5	15.0	17.4	14.0
C–O/C–Br (286.4 eV)	4.4	4.9	1.1	4.0
C=O (288 eV)	4.8	4.7	5.8	5.1
O–C=O (289-290 eV)	7.8	6.4	8.3	7.1
shake-up satellites	2.4	2.3	2.5	1.9

**Table 4:** XRD parameters for selected MWCNT-Br_n_ samples.

Bromination Time (Days)	Lattice Parameters (Å)	Interlayer Distances (Å)	Space Group
0	a = 2.523 c = 6.958	3.479	P6_3_/mmc
7	a = 2.625 c = 8.620	4.310	P6_3_/mmc
10	a = 2.652 c = 9.258	4.629	P6_3_/mmc
14	a = 2.675 c = 9.420	4.710	P6_3_/mmc

**Table 5:** Raman shift for MWCNT before and after the 10 days bromination.

Type of Vibration	Raman Shift (cm^−1^)		
	Literature Value	MWCNT Native	MWCNT–Br 10
D	approx. 1350 [56,57]	1336	1345
G	approx. 1580 [56,57]	1580	1592
G’	approx. 2700 [56,57] (overtone of D-vibration)	2670	2687

## Data Availability

Data is contained within the article.
